# A real-world pharmacovigilance analysis for transthyretin inhibitors: findings from the FDA adverse event reporting database

**DOI:** 10.3389/fphar.2024.1368244

**Published:** 2024-05-30

**Authors:** Yuan Liu, Hao Li, Cheng Hu, Li Tan, Ping Yin, Zhihao Li, Shuangshan Zhou, Li Su

**Affiliations:** ^1^ Department of Cardiology, The Second Affiliated Hospital, Chongqing Medical University, Chongqing, China; ^2^ Second Clinical College, Chongqing Medical University, Chongqing, China

**Keywords:** patisiran, vutrisiran, inotersen, FAERS, pharmacovigilance analysis

## Abstract

**Objective:**

The purpose of this study is to investigate the drug safety of three Transthyretin (TTR) inhibitors in the real world using the United States Food and Drug Administration Adverse Event Reporting System (FAERS) database.

**Methods:**

This study extracted reports received by the FAERS database from the first quarter of 2018 to the third quarter of 2023 for descriptive analysis and disproportionality analysis. Safety signal mining was conducted at the Preferred Term (PT) level and the System Organ Class (SOC) level using reporting odds ratio (ROR). The characteristics of the time-to-onset curves were analyzed using the Weibull Shape Parameter (WSP). The cumulative incidence of TTR inhibitors was evaluated using the Kaplan-Meier method. Subgroup analyses were conducted based on whether the reporter was a medical professional.

**Results:**

A total of 3,459 reports of adverse events (AEs) caused by TTR inhibitors as the primary suspect (PS) drug were extracted. The top three reported AEs for patisiran were fatigue, asthenia, and fall, with the most unexpectedly strong association being nonspecific reaction. The top three reported AEs for vutrisiran were fall, pain in extremity and malaise, with the most unexpectedly strong association being subdural haematoma. The top three reported AEs for inotersen were platelet count decreased, blood creatinine increased, and fatigue, with the most unexpectedly strong association being blood albumin decreased. Vitamin A decreased, arthralgia, and dyspnea were the same AEs mentioned in the drug labels of all three drugs, while malaise and asthenia were the same unexpected significant signals. This study offers evidence of the variability in the onset time characteristics of AEs associated with TTR inhibitors, as well as evidence of differences in adverse event reporting between medical professionals and non-medical professionals.

**Conclusion:**

In summary, we compared the similarities and differences in drug safety of three TTR inhibitors in the real world using the FAERS database. The results indicate that not only do these three drugs share common AEs, but they also exhibit differences in drug safety profiles. This study contributes to enhancing the understanding of medical professionals regarding the safety of TTR inhibitors.

## Introduction

Transthyretin amyloidosis (ATTR) is a disease characterized by the abnormal deposition of transthyretin in multiple tissues and organs due to misfolding (Castaño et al., 2015; Wechalekar et al., 2016). ATTR is marked by its low prevalence, multisystem involvement, and the challenges in clinical and differential diagnosis (Maurer et al., 2019; Adams et al., 2021). The clinical phenotypes primarily include transthyretin amyloid polyneuropathy (ATTR-PN) and transthyretin amyloid cardiomyopathy (ATTR-CM) (Adams et al., 2019; Maurer et al., 2019; Ruberg et al., 2019). Approximately 70% of ATTR patients present with peripheral neuropathy (Siddiqi and Ruberg, 2018). Neuropathies in ATTR patients may include carpal tunnel syndrome, lumbar spinal stenosis, small fiber neuropathy, and autonomic dysfunction. As the disease progresses, patients may also experience loss of reflexes, reduced motor skills, and muscle weakness (Cortese et al., 2017; Barge-Caballero et al., 2019; Carr et al., 2019). Accurate global prevalence estimates for ATTR remain elusive, however, recent research has broadened our understanding. A retrospective study estimated the global prevalence of ATTR-PN at 10,186 (range 5,526–38,468) (Schmidt et al., 2018). Another study highlighted geographical variations in the prevalence of certain ATTR genotypes. Specifically, the Val30Met genotype is most prevalent in endemic countries, while genotypes in non-endemic countries are primarily categorized as “other" (Waddington-Cruz et al., 2019). The average survival period after the onset of ATTR ranges from 6 to 12 years (Adams et al., 2021). Previously, treatment options for ATTR were limited. Apart from symptomatic treatment for neuropathy, heart failure, and arrhythmias, liver transplantation was the only effective treatment (Sekijima, 2015; Maurer et al., 2018). However, liver transplantation has limited efficacy in halting disease progression and is associated with high costs, surgical complications, and transplant-related rejection reactions in clinical practice (Carvalho et al., 2015). In recent years, the introduction of TTR inhibitors has provided new treatment options for ATTR patients. They primarily function by inhibiting the translation process of TTR mRNA. As of the third quarter of 2023, only patisiran (approval time: 2018), vutrisiran (approval time: 2022), and inotersen (approval time: 2018), three TTR inhibitors, have been approved by the FDA for the treatment of adult hereditary ATTR-PN (Benson et al., 2018; Maurer et al., 2018; Park et al., 2019; Darrow et al., 2020; Nie et al., 2023).

All TTR inhibitors received Orphan Drug Designation (ODD) or Fast Track designation during their FDA approval process. In previous limited clinical studies, AEs to patisiran included upper respiratory tract infections, infusion-related reactions, indigestion, dyspnea, arthralgia, muscle spasms, etc. (Adams et al., 2018; Maurer et al., 2023), while inotersen’s AEs included glomerulonephritis, thrombocytopenia, injection site reactions, nausea, headaches, etc. (Benson et al., 2018). AEs to amvuttra included injection AEs, dyspnea, arthralgia, and vitamin A deficiency (Habtemariam et al., 2021). It is important to note that the FDA has warned about inotersen causing thrombocytopenia and glomerulonephritis through a black box warning. It is noteworthy that due to the rarity of the disease, challenges in diagnosis, and limited follow-up time in clinical trials, large-scale clinical studies investigating the drug safety of TTR inhibitors remain insufficient, making rare drug-related AEs difficult to observe. Therefore, post-marketing surveillance of drug-related AEs for TTR inhibitors is crucial.

The FAERS database is a project operated by the FDA to identify potential correlations between drugs and AEs within the scope of post-marketing drug safety surveillance (FAERS, 2023). This public platform encourages medical professionals, patients, pharmaceutical companies, and the public to report AEs through the MedWatch program. Earlier studies have utilized the FAERS database for assessing the real-world safety of drugs. For instance, Lindsy Pang and others evaluated post-marketing AEs of non-stimulant attention deficit hyperactivity disorder medications using the FAERS database. Natalia Gonzalez Caldito and others analyzed the differences in reported AEs between rituximab and ocrelizumab using the FAERS database (Caldito et al., 2021; Pang and Sareen, 2021).

The FAERS database is updated quarterly and currently holds a vast dataset of over ten million reports. The purpose of our study is to extract reports of AEs related to TTR inhibitors in the real world from the extensive data of the FAERS database and further conduct retrospective pharmacovigilance research based on these critical data. The results of the study will be beneficial for the clinical practice of TTR inhibitors, providing valuable references for medical professionals.

## Materials and methods

### Data source

We conducted a real-world pharmacovigilance study on three TTR inhibitors using the latest data from the FAERS database. Since the FDA first approved the TTR inhibitor patisiran in 2018, we extracted report data from the FAERS database from the first quarter of 2018 to the third quarter of 2023. The analysis utilized four sub-databases: DEMO, DRUG, REAC, and THER, they respectively provide demographic clinical characteristics (such as gender, age, reporting time), medication usage information (drug names, routes of administration, dosages), adverse event details (names of AEs), and medication date information (start and end dates of drug treatment).

### Data preprocess

**FIGURE 1:**
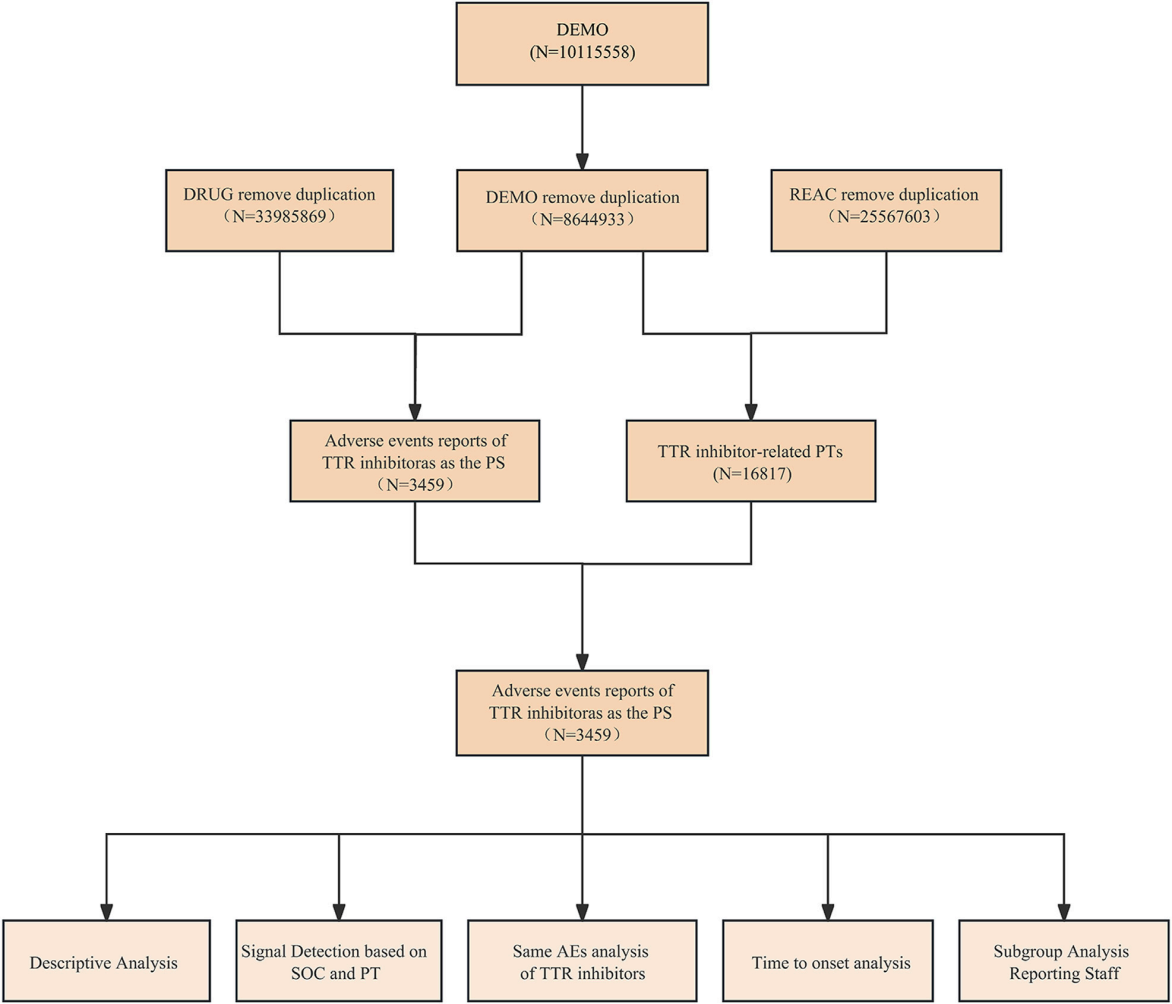
Flow diagram of this study.

To enhance the reliability of our research, we preprocessed the initially obtained data, with the detailed process illustrated in Figure 1. Firstly, we identified patisiran, vutrisiran, and inotersen as target drugs. Considering that the FAERS database allows the use of multiple names for drugs when reporting AEs, both the drug and brand names of these three drugs were used for retrieval in the FAERS database to avoid missing related data. We obtained reports of AEs caused by TTR inhibitors as the primary suspect (PS) drug from the first quarter of 2018 to the third quarter of 2023 by searching for patisiran (ALN-TTR02, onpattro, patisiran sodium), vutrisiran (amvuttra, vutrisiran sodium), and inotersen (inotersen sodium, tegsedi) in the FAERS database. Secondly, we removed duplicates based on CASEID, FDA_DT, and PRIMARYID in the FAERS database, as demonstrated in Table 1 (Yu et al., 2023). Finally, we analyzed the data based on the hierarchical structure of the International Medical Dictionary (MedDRA) version 26.1, from the SOC and the PT levels (Brown, 2002; Brown, 2003). SOC is the top-level classification in MedDRA, typically involving a particular system or organ of the body. PT refers to the preferred term for specific symptoms or diagnoses. Furthermore, we manually reviewed and revised the names of SOCs and PTs in the FAERS database according to MedDRA.

**TABLE 1:** Example of FAERS database duplicate report removal rule.

CASEID	FDA_DT	PRIMARYID	Delete or save
2,751,007	40,064,207	51,321,061	DELETE
2,751,007	40,064,345	51,364,244	SAVE
2,720,901	40,064,112	26,542,012	DELETE
2,720,901	40,064,112	27,532,429	SAVE

### Descriptive analysis

Following the aforementioned process, we examined the final dataset related to TTR inhibitors and summarized the clinical characteristics of the population, including gender, age group, reporter, and year of report.

### Disproportionality analysis

We first examined the adverse event data of the three TTR inhibitors at the SOC level, calculating the number of reports for various AEs, the reporting odds ratio (ROR), and its 95% Confidence Interval (CI) for each drug. Subsequently, at the PT level, we ranked the AEs based on the number of reports, generating a list of the top 50 AEs for each of the three TTR inhibitors, and then calculated their ROR and 95% CI. ROR is used to assess the correlation between TTR inhibitors and specific AEs and has been successfully applied in previous studies based on the FAERS database, with the formula and standards presented in Supplementary Table S1 (Ooba and Kubota, 2010; Musialowicz et al., 2023). During our analysis at the PT level, we excluded data related to outcomes, injections or infusions, and clinical characteristics of ATTR-CM and ATTR-PN, except for data on arrhythmias. When analyzing at the PT level, we excluded data related to outcomes, injections or infusions, and symptoms caused by the disease itself. Additionally, we summarized the same AEs already listed in the drug labels of the three TTR inhibitors and the same unexpected significant signals obtained from this study. In this study, an unexpected significant signal is defined as: a specific adverse event at the PT level that meets the ROR algorithm standards and is not mentioned in the most recent version of the drug labels to date.

### Time to onset analysis

Time-to-onset was determined based on the interval between EVENT_DT (date of adverse event occurrence) and START_DT (date of initiating drug treatment) in the THER sub-database. We excluded reports with inaccurate dates, missing dates, and those where the start date of drug treatment was after the date of the adverse event occurrence. The assessment utilized median, quartiles, and the WSP test. The WSP test identifies the rate of change in the incidence of AEs, with the scale parameter α and shape parameter β obtained from the test being critical in determining the scale and shape of the distribution function. Based on the shape parameter β, the risk in a reference population can be assessed, categorized as follows: when β < 1 and its 95% CI < 1, it is considered that the risk of drug-related AEs decreases over time (early failure-type profile); when β is equal to or close to 1 and its 95% CI includes 1, it is considered that the risk of drug-related AEs occurs continuously over time (random failure-type profile); when β > 1 and its 95% CI > 1, it is considered that the risk of drug-related AEs increases over time (wear-out failure-type profile) (Cornelius et al., 2012). Additionally, the Kaplan-Meier method was used to plot the cumulative incidence of AEs related to the three drugs in figures for comparison purposes.

### Subgroup analyses

We conducted subgroup analyses based on whether the reporter was a medical professional, with the following method: extracting reports of AEs related to the three TTR inhibitors reported by both healthcare and non-medical professionals; at the PT level, selecting the top 50 AEs by number of reports; and calculating the ROR value and its 95% CI for signal mining.

In this study, ROR was calculated by comparison with the FAERS database. Data processing and statistical analysis were conducted using Microsoft Excel 2019 and R software version 4.3.1. Tables were created using Microsoft Word 2019, and figures were produced using R software version 4.3.1.

## Results

### Descriptive analysis

Between the first quarter of 2018 and the third quarter of 2023, the FAERS database received a total of 10,115,558 case reports. After eliminating duplicate reports, there were 8,644,933 patient cases with 25,567,603 reported AEs. Within these data, there were 3,459 reports of AEs caused by TTR inhibitors as the PS drugs. The summarized clinical characteristic information can be seen in Table 2.

**TABLE 2:** Clinical Characteristics of Reports with transthyretin inhibitors from the FAERS Database.

	Patisiran	Vutrisiran	Inotersen
Total number of reports	2158	258	1,043
Sex			
Female	213 (9.9%)	0	248 (23.8%)
Male	400 (18.5%)	0	409 (39.2%)
Missing	1,545 (71.6%)	258 (100%)	386 (37.0%)
Age (years)			
<18	0	0	0
18–65	94 (4.4%)	0	237 (22.7%)
>65	153 (7.1%)	0	379 (36.4%)
Missing	1911 (88.6%)	258 (100%)	427 (40.9%)
Reporting Staff			
Medical professionals	1,275 (59.1%)	89 (34.5%)	623 (59.7%)
Non-medical professionals	866 (40.1%)	169 (65.5%)	419 (40.2%)
Missing	17 (0.8%)	0	1 (0.1%)
Reporting year			
2018	23 (1.1%)	0	0
2019	337 (15.6%)	0	64 (6.1%)
2020	478 (22.2%)	0	268 (25.7%)
2021	456 (21.1%)	0	156 (15.0%)
2022	511 (23.7%)	18 (7.0%)	216 (20.7%)
2023	353 (16.3%)	240 (93.0%)	339 (32.5%)

In reports related to patisiran, the proportion of males (18.5%) was higher than that of females (9.9%), with 71.6% of reports having an unknown gender; the largest age group was over 65 years (7.1%), followed by 18–65 years (4.4%), with no reports under 18 years and 88.6% having an unknown age; reports uploaded by medical professionals accounted for 59.1% compared to 40.1% by non-medical professionals, with 0.8% unknown reporters; the year with the most reports was 2022 (23.7%), followed by 2020 (22.2%), 2021 (21.1%), 2023 (16.3%), 2019 (15.6%), and 2018 (1.1%).

In reports related to vutrisiran, all reports had unknown gender and age; reports uploaded by non-medical professionals accounted for 65.5%, higher than those by medical professionals (34.5%), with no reports having an unknown reporter; the year with the most reports was 2023 (93.0%), followed by 2022 (7.0%).

In reports related to inotersen, the proportion of males (39.2%) was higher than that of females (23.8%), with 37.0% of reports having an unknown gender; the largest age group was over 65 years (36.4%), followed by 18–65 years (22.7%), with no reports under 18 years and 40.9% having an unknown age; reports uploaded by medical professionals accounted for 59.7% compared to 40.2% by non-medical professionals, with 0.1% unknown reporters; the year with the most reports was 2023 (32.5%), followed by 2020 (25.7%), 2022 (20.7%), 2021 (15.0%), and 2019 (6.1%).

### Disproportionality analysis

At the SOC level, the number of reports and signal strength of AEs related to the three TTR inhibitors are presented in Supplementary Figure S1–S3. We found that AEs related to patisiran involved 26 organ systems. Significant SOC meeting the criteria included social circumstances, ear and labyrinth disorders, metabolic and nutritional disorders, various types of injuries, poisonings and procedural complications, general disorders and administration site conditions, vascular and lymphatic disorders, various neurological disorders, cardiac disorders, and various surgeries and medical procedures. AEs related to vutrisiran involved 23 organ systems, with significant SOC including various musculoskeletal and connective tissue disorders, cardiac disorders, various neurological disorders, and various surgeries and medical procedures. AEs related to inotersen involved 26 organ systems, with significant SOC including social circumstances, renal and urinary disorders, and various investigations.

**FIGURE 2:**
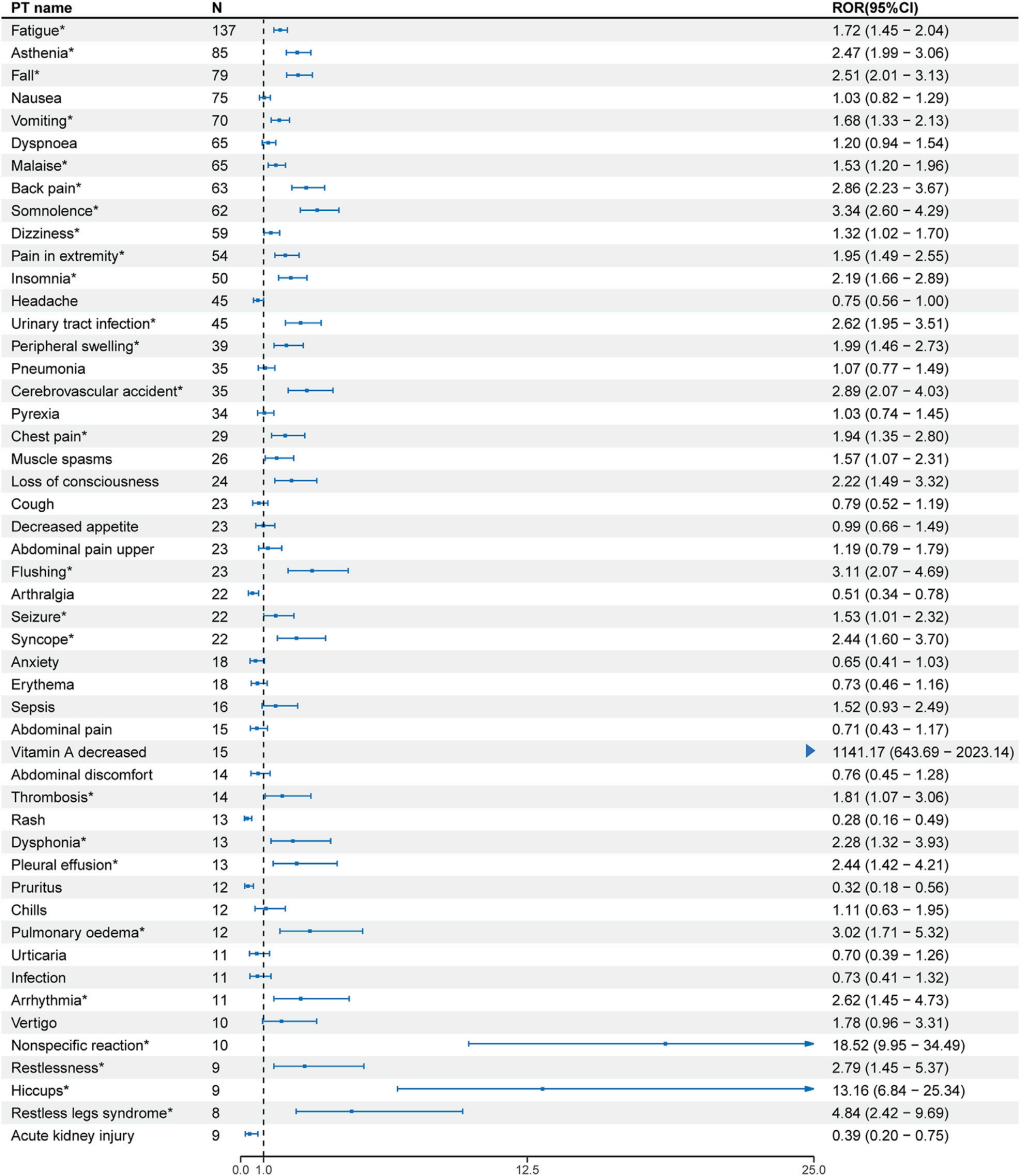
Signal strength of the top 50 AEs for patisiran ranked by report frequency at the preferred term (PT) Level in the FDA Adverse Event Reporting System (FAERS) N:number of drug-related AEs *: mentioned in the drug label and ROR_025_ > 1 and N ≥ 2.

**FIGURE 3:**
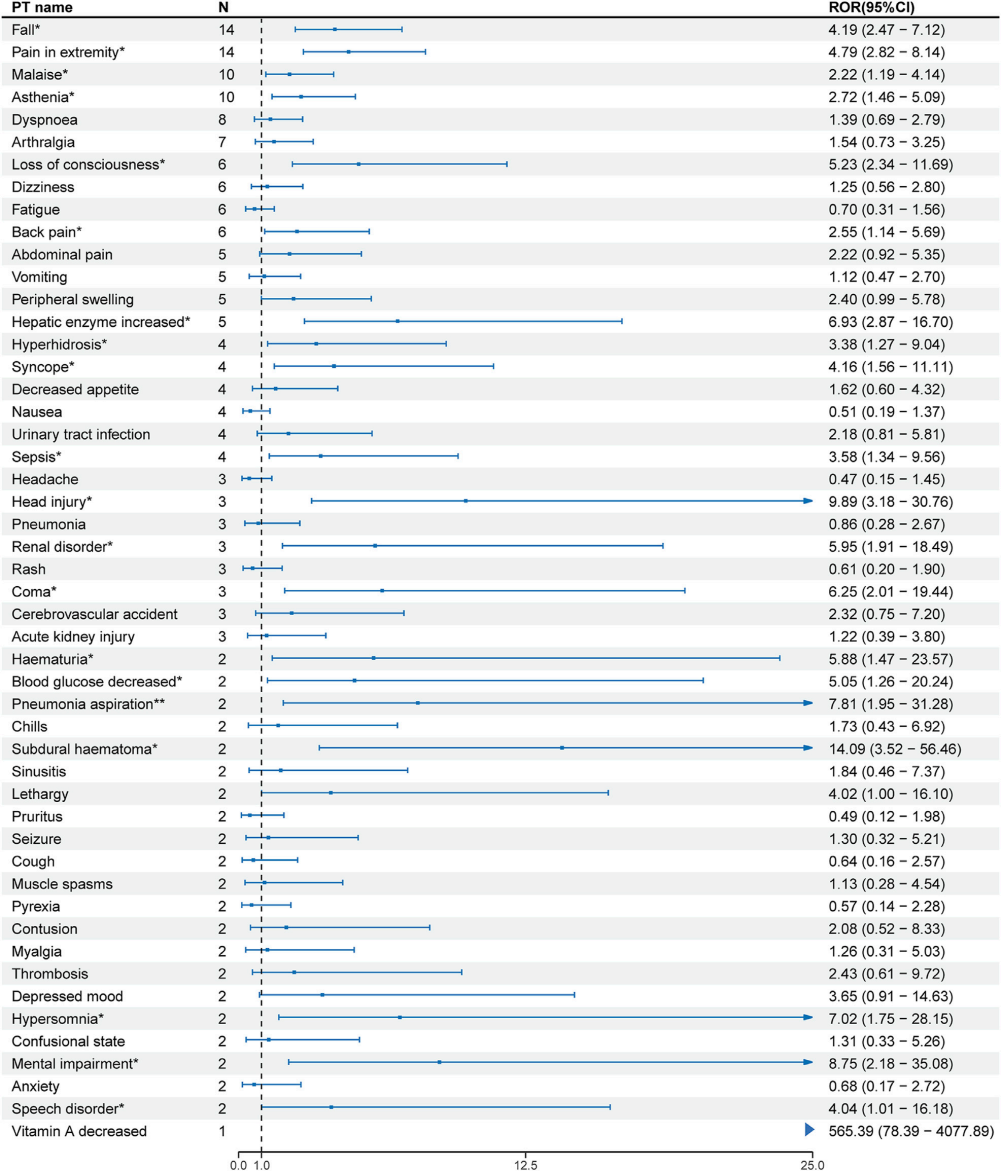
Signal strength of the top 50 AEs for vutrisiran ranked by report frequency at the preferred term (PT) Level in the FDA Adverse Event Reporting System (FAERS) N:number of drug-related AEs *: mentioned in the drug label and ROR_025_> 1 and N ≥ 2.

**FIGURE 4:**
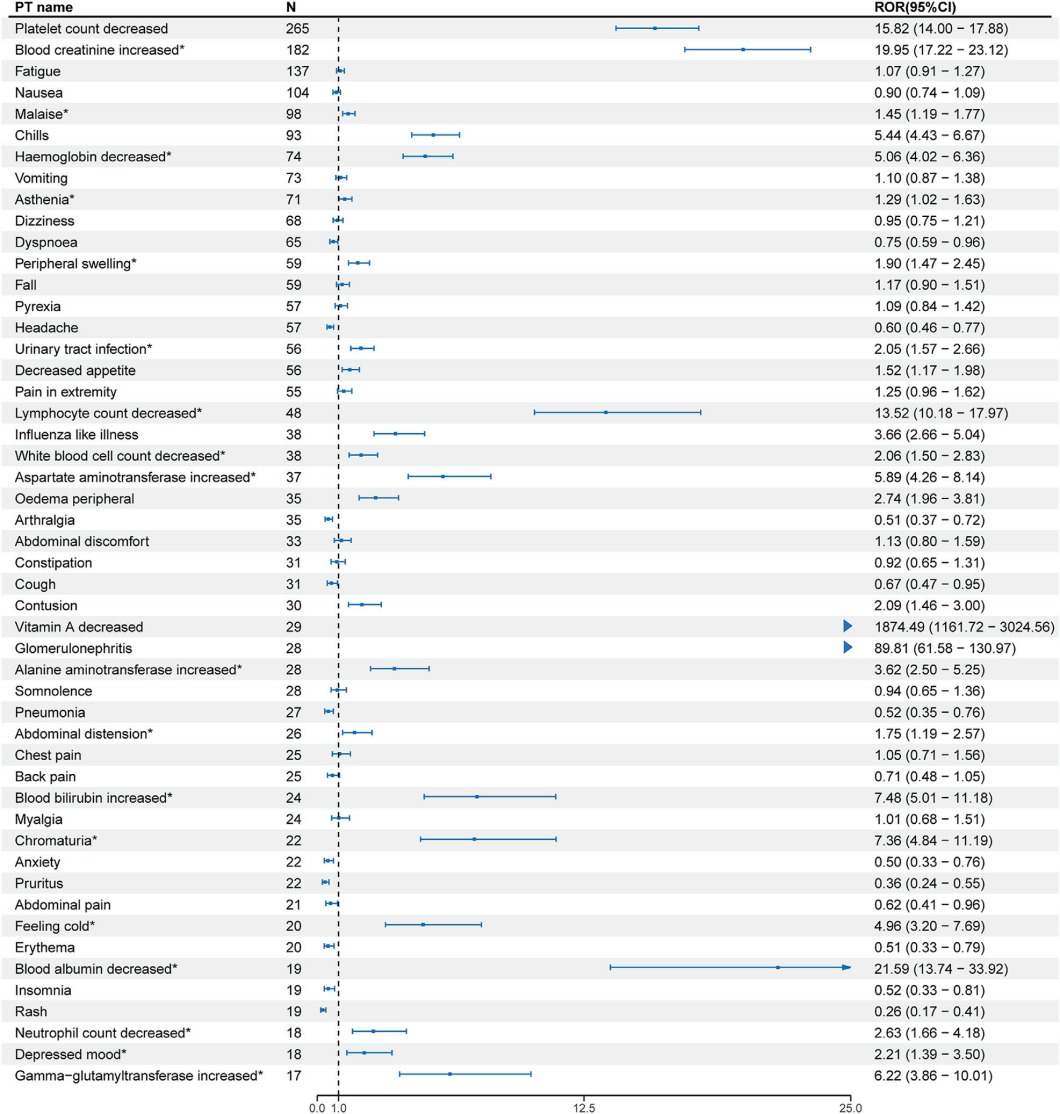
Signal strength of the top 50 AEs for inotersen ranked by report frequency at the preferred term (PT) Level in the FDA Adverse Event Reporting System (FAERS) N:number of drug-related AEs *: mentioned in the drug label and ROR_025_ > 1 and N ≥ 2.

At the PT level, the number of reports and signal strength of the top 50 most common AEs related to the three TTR inhibitors are shown in Figures 2–4. The top 10 reported AEs related to patisiran were fatigue, asthenia, fall, nausea, vomiting, dyspnoea, malaise, back pain, somnolence, and dizziness. A total of 29 AEs were significant signals, with vitamin A decreased and muscle spasms mentioned in the drug label, and the other 27 as unexpected significant signals. The top 10 reported AEs related to vutrisiran were fall, pain in extremity, malaise, asthenia, dyspnoea, arthralgia, loss of consciousness, dizziness, fatigue, and back pains, with 19 being significant signals not mentioned in the latest drug label. The top 10 reported AEs related to inotersen were platelet count decreased, blood creatinine increased, fatigue, nausea, malaise, chills, haemoglobin decreased, vomiting, asthenia, and dizziness, with 25 being significant signals, including decreased appetite, contusion, oedema peripheral, influenza like illness, chills, platelet count decreased, glomerulonephritis, and vitamin A decreased mentioned in the drug label, and the other 19 as unexpected significant signals.

We compared the AEs mentioned in the latest version of the drug labels of the three TTR inhibitors with the unexpected significant signals obtained from this study, finding that vitamin A decreased, arthralgia, and dyspnea were the same AEs mentioned in the drug labels, with malaise and asthenia as the same unexpected significant signals.

### Time to onset analysis

**FIGURE 5:**
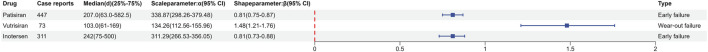
Weibull shape parameter test for AEs associated with transthyretin inhibitors CI:confidence interval.

**FIGURE 6:**
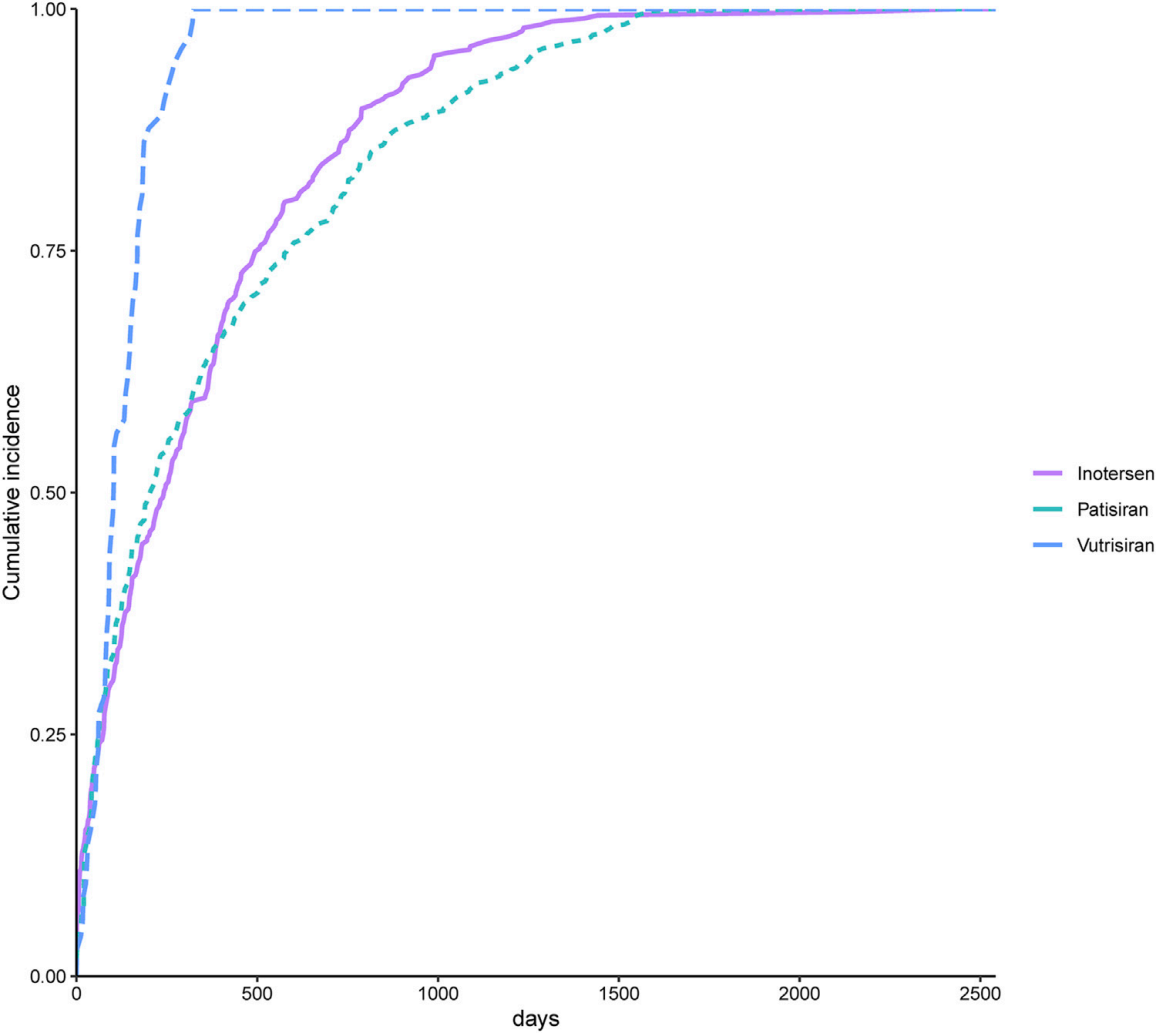
Comparison between cumulative incidences of AEs between patients treated with transthyretin inhibitors.

We included reports of 447 AEs related to patisiran, 73 AEs related to vutrisiran, and 311 AEs related to inotersen for time-to-onset analysis, with detailed results presented in Figure 5. The median time-to-onset for AEs related to patisiran was 207 days (63–582.5 days), with the WSP test’s β and its 95% CI being 0.81 (0.75–0.87), indicating an early failure-type profile. The median time-to-onset for AEs related to vutrisiran was 103 days (61–169 days), with the WSP test’s β and its 95% CI being 1.48 (1.21–1.76), indicating a wear-out failure-type profile. The median time-to-onset for AEs related to inotersen was 242 days (75–500 days), with the WSP test’s β and its 95% CI being 0.81 (0.73–0.88), indicating an early failure-type profile. Figure 6 shows the cumulative incidence of AEs related to the three TTR inhibitors. The percentage of AEs occurring within 30 days of starting patisiran treatment was 14.3%, and within 180 days was 46.8%. For vutrisiran, the percentage of AEs within 30 days of treatment was 12.3%, and within 180 days was 79.5%. For inotersen, the percentage of AEs within 30 days of treatment was 15.4%, and within 180 days was 44.4%.

### Subgroup analyses

Our subgroup analysis results based on whether the reporter was a medical professional are presented in Supplementary Table S2–S4. In reports of AEs related to patisiran, common significant signals included fatigue, insomnia, pain in extremity, asthenia, fall, back pain, chest pain, somnolence, urinary tract infection, cerebrovascular accident, vitamin A decreased, peripheral swelling, and syncope. Unique significant signals reported by medical professionals included flushing, seizure, pleural effusion, restlessness, dysphonia, pulmonary edema, influenza-like illness, thrombosis, hiccups, and restless legs syndrome. Unique significant signals reported by non-medical professionals included muscle spasms, muscle weakness, loss of consciousness, sepsis, vertigo, limb discomfort, and taste disorder.

In reports of AEs related to vutrisiran, pain in extremity was a common significant signal. Unique significant signals reported by medical professionals included hepatic enzyme increased, sepsis, malaise, abdominal pain, and syncope. Unique significant signals reported by non-medical professionals included fall, asthenia, loss of consciousness, peripheral swelling, hyperhidrosis, urinary tract infection, head injury, renal disorder, limb discomfort, subdural hematoma, coma, hypersomnia, and mental impairment.

In reports of AEs related to inotersen, common significant signals included platelet count decreased, blood creatinine increased, malaise, chills, urinary tract infection, haemoglobin decreased, contusion, vitamin A decreased, feeling cold, and lymphocyte count decreased. Unique significant signals reported by medical professionals included decreased appetite, peripheral swelling, alanine aminotransferase increased, aspartate aminotransferase increased, influenza-like illness, glomerulonephritis, blood bilirubin increased, abdominal distension, blood lactate dehydrogenase increased, gamma-glutamyltransferase increased, blood alkaline phosphatase increased, and depressed mood. Unique significant signals reported by non-medical professionals included pyrexia, asthenia, vomiting, chest pain, red blood cell count decreased, anemia, aspartate aminotransferase abnormal, alanine aminotransferase abnormal, and pulmonary oedema.

## Discussion

TTR inhibitors are important therapeutic drugs for patients with ATTR. TTR inhibitors developed based on Small interfering RNA (siRNA) or antisense oligonucleotide (ASO) have been proven to be highly effective in blocking the expression of TTR in human liver in several clinical trials, with drugs based on siRNA including patisiran and vutrisiran, and the drug based on ASO being inotersen (Aimo et al., 2022). In this study, we extracted reports of AEs related to TTR inhibitors from the FAERS database and conducted drug safety signal mining at both SOC and PT levels, identifying several significant signals not mentioned in drug labels. To our knowledge, this is the first study to conduct a real-world pharmacovigilance analysis on multiple TTR inhibitors.

The results indicate that patisiran, inotersen, and vutrisiran have different AEs spectra in the real world. Ranked by report numbers, the top three AEs for patisiran are fatigue, asthenia, and fall; for inotersen, they are platelet count decreased, blood creatinine increased, and fatigue; and for vutrisiran, they are fall, pain in extremity and malaise.

Patisiran is the first TTR inhibitor approved by the FDA, specifically inhibiting the synthesis of TTR in the liver (Adams et al., 2018). After excluding AEs related to death, outcomes, and symptoms caused by the disease itself at the PT level, fatigue is the most reported adverse event for patisiran, with 137 reports and not mentioned in the drug label. siRNA drugs may cause off-target reactions in clinical applications, leading to drug toxicity reactions. We speculate the potential mechanism is that siRNA drugs and their delivery carriers might activate the innate immune system, leading to inflammatory responses, which could cause systemic fatigue and weakness (Jackson and Linsley, 2010). A meta-analysis using siRNA to treat acute intermittent porphyria also found fatigue as a common adverse event (Patel et al., 2023).

Inotersen is a 2′- O-methoxyethyl-modified antisense oligonucleotide. Renal and urinary disorders were significant signals at the SOC level, and increased blood creatinine was the second most reported significant signal at the PT level, with glomerulonephritis ranking 30th. The FDA has warned about inotersen causing glomerulonephritis in a boxed warning on its label, and our study re-emphasizes the need to be vigilant about inotersen’s renal toxicity in clinical practice. Notably, thrombocytopenia is the most reported significant signal at the PT level. Thrombocytopenia is an important adverse event, and the FDA has also warned about it in a boxed warning on the drug label, consistent with our study results. A clinical study of 172 adult patients not only found thrombocytopenia as the most common severe adverse event of inotersen but also detected anti-platelet IgG antibodies shortly before or at the time of severe thrombocytopenia occurrence. The reaction between anti-platelet IgG antibodies and EDTA can cause platelet aggregation, leading to inexplicable platelet measurement results, which delays diagnosis and treatment (Benson et al., 2018). We speculate that ASO may be one of the causes of thrombocytopenia induced by inotersen. Clinical trials of two other ASO drugs also found thrombocytopenia as a drug-related adverse event (Shamsudeen and Hegele, 2022; Thornton et al., 2023). According to the results of one drug trial, the possible mechanisms of ASO-induced thrombocytopenia include ASO forming polymers, the nucleic acid part of the polymer interacting with plasma proteins and platelets to form aggregates, and platelets bound to aggregates being activated, leading to platelet aggregation and reduction in circulating platelet count (Harada et al., 2023).

Vutrisiran is a subcutaneously administered transthyretin-directed siRNA. Since vutrisiran was the latest of the three drugs to be approved (2022), there are fewer safety studies and reports related to vutrisiran in this study compared to the other two drugs. Through pharmacovigilance analysis, we found fall and asthenia as common significant signals with patisiran, with the activation of immune responses and inflammatory reactions by siRNA being a possible mechanism for both causing fall and asthenia (Jackson and Linsley, 2010).

The same AEs mentioned in the drug labels of all three TTR inhibitors include vitamin A decreased, arthralgia, and dyspnea. Vitamin A decreased ranked 33rd in patisiran-related reports, 50th in vutrisiran-related reports, and 29th in inotersen-related reports. TTR is a 55 kDa tetramer transport protein consisting of four identical subunits of 127 amino acids (Li and Buxbaum, 2011). Human TTR can carry retinol-binding protein bound to retinol, and to transport retinol, TTR needs to form a tetramer and bind to retinol-binding protein (Vieira and Saraiva, 2014). The TTR-RBP complex is a very stable form of retinol transport, safely transporting it to target tissues without being filtered and degraded by the kidneys (Goodman, 1984; Noy et al., 1992). TTR inhibitors block the formation of TTR protein, slowing the progression of ATTR disease, and also block the transport pathway of retinol, preventing retinol from forming a stable transport structure and possibly being degraded and excreted in the blood circulation, ultimately leading to vitamin A deficiency in patients. Previous animal experiments found that TTR knockout (KO) mice compared to wild-type (WT) animals showed significantly lower serum retinol levels, indicating TTR’s important role in maintaining normal vitamin A levels in the body (Episkopou et al., 1993). Consistent with FDA recommendations, our study results also emphasize the need for patients using TTR inhibitors to supplement and monitor vitamin A to avoid related diseases such as night blindness.

Previous clinical trial results with TTR inhibitors support arthralgia as a common adverse event for all three TTR inhibitors. One clinical trial found a higher frequency of arthralgia in patients treated with patisiran compared to the placebo group. Another clinical trial found patients tolerated vutrisiran well, with the only two more common AEs compared to the placebo group being limb pain and arthralgia. A clinical trial of inotersen also found a 5% higher frequency of arthralgia in the inotersen group compared to the placebo group (Maurer et al., 2023; Nie et al., 2023). However, the mechanism of TTR inhibitors causing arthralgia is currently unclear.

In our study, asthenia and malaise are the same unexpected significant signals for all three TTR inhibitors, both being nonspecific symptoms. Since ATTR is a multisystem disease, the progression of the disease may also lead to asthenia and malaise, so more research is needed to clarify the relationship between TTR inhibitors and asthenia and malaise.

The WSP test showed that AEs related to vutrisiran have a wear-out failure-type profile, indicating that the risk of AEs related to vutrisiran increases over time. The WSP test also showed that AEs related to patisiran and inotersen have an early failure-type profile, indicating that the risk of drug-related AEs decreases over time. The pharmacokinetic characteristics of the three drugs may help explain the differences in the characteristics of adverse event onset. The median time to onset of AEs for vutrisiran is shorter than the other two drugs. It should be noted that vutrisiran has been on the market for a shorter time, and those AEs with longer onset times have not yet occurred and been reported, which may cause bias in the statistical results.

In our subgroup analysis based on whether the reporter was a medical professional, we found differences in AEs for different subgroups of TTR inhibitors. Medical professionals are more precise in classifying AEs, while non-medical professionals are more general. For example, in the subgroup analysis results for inotersen, both subgroups reported drug-induced liver function abnormalities, with medical professionals reporting them more accurately as elevated AST or ALT, while non-medical professionals reported them as abnormal AST or ALT. Moreover, at the PT level, AEs reported by non-medical professionals are mainly related to symptoms, while disease-related AEs requiring medical knowledge are more often reported by medical professionals. For example, in our study, 82.1% of reports of glomerulonephritis caused by inotersen and 75.0% of reports of restless legs syndrome caused by patisiran were reported by medical professionals. This shows that non-medical professionals have limitations in judging drug-related AEs compared to medical professionals.

Our study also has some limitations. Firstly, pharmacovigilance analysis through the FAERS database can only indicate the correlation between a specific drug and specific AEs, and cannot infer causality. Secondly, the FAERS database has incomplete reporting information, such as missing gender and age in vutrisiran-related reports, and a high proportion of missing gender or age in reports related to the other two TTR inhibitors. Therefore, in this study, we only conducted subgroup analysis based on whether the reporter was a medical professional. Finally, the FAERS database has some inherent biases, such as a greater likelihood of not reporting AEs that are too mild or too complex. Due to the voluntary nature of the FAERS database, it may lead to inconsistent reporting, including an increased propensity to report AEs considered related to a given drug. Despite these limitations, the FAERS database provides an opportunity to assess the drug safety of drugs used to treat rare diseases in the real world.

## Conclusion

In summary, by utilizing the extensive data from the FAERS database, we compared the similarities and differences in drug safety of three TTR inhibitors in the real world. The results show that not only do these drugs share common AEs, but they also exhibit differences in terms of drug safety. This study contributes to enhancing medical professionals’ understanding of the safety of TTR inhibitors and provides valuable insights for clinical practice. Future pharmacological epidemiology studies are needed to further explore the drug safety of TTR inhibitors.

## Data Availability

Publicly available datasets were analyzed in this study. This data can be found here: https://fis.fda.gov/extensions/FPD-QDE-FAERS/FPD-QDE-FAERS.html.
