# Supplementary material for: A real-world pharmacovigilance analysis for transthyretin inhibitors: findings from the FDA adverse event reporting database
Source: Front Pharmacol. 2024 May 30;15:1368244. doi: 10.3389/fphar.2024.1368244 (PMC11169801; doi:10.3389/fphar.2024.1368244)
Supplement: Supplementary file 1 [file DataSheet1.docx]

Supplementary Material

# Supplementary Figures and Tables

## Supplementary Figures


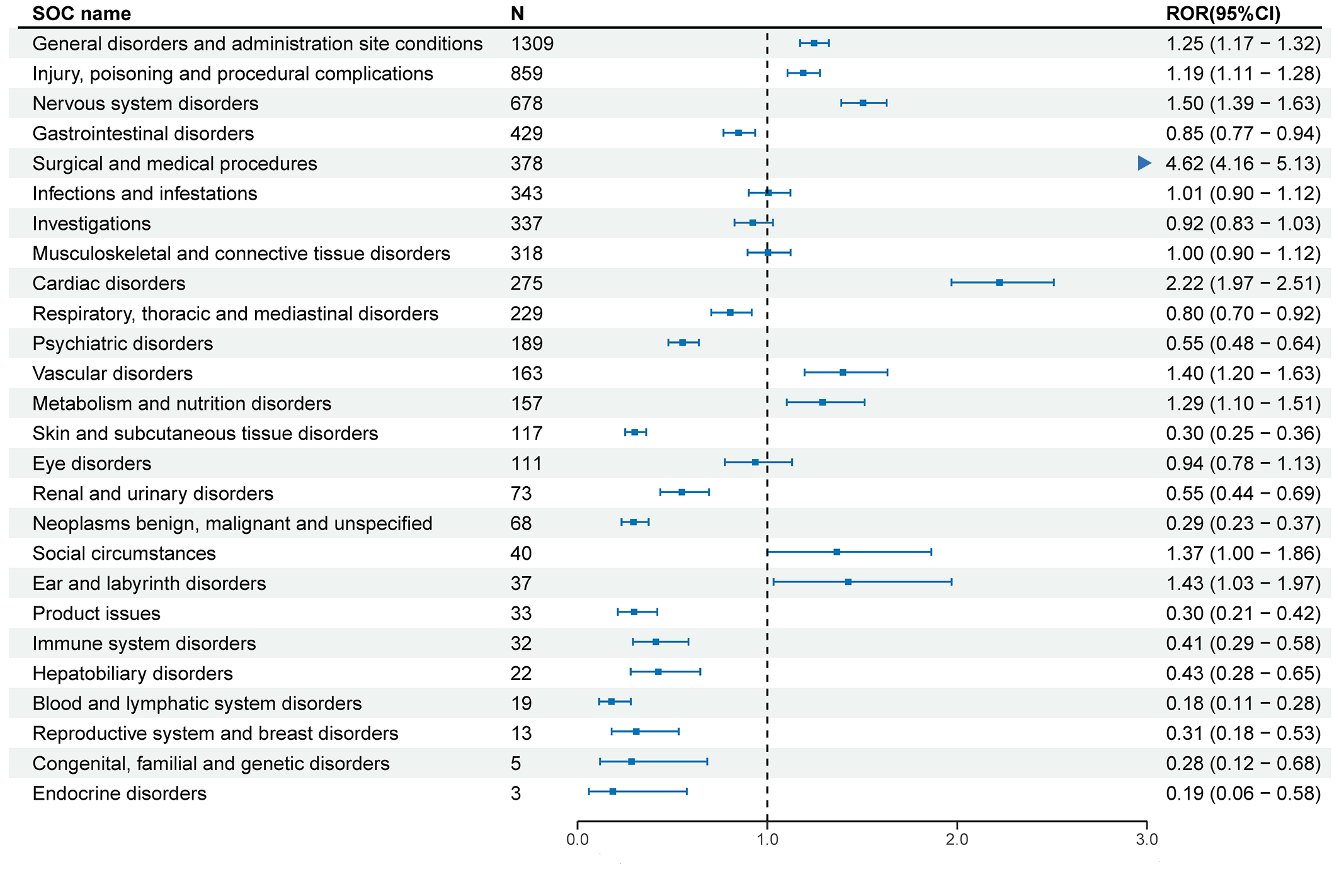


**Supplementary Figure 1**:Signal strength of AEs of Patisiran at the System Organ Class (SOC) level in FDA Adverse Event Reporting System (FAERS)


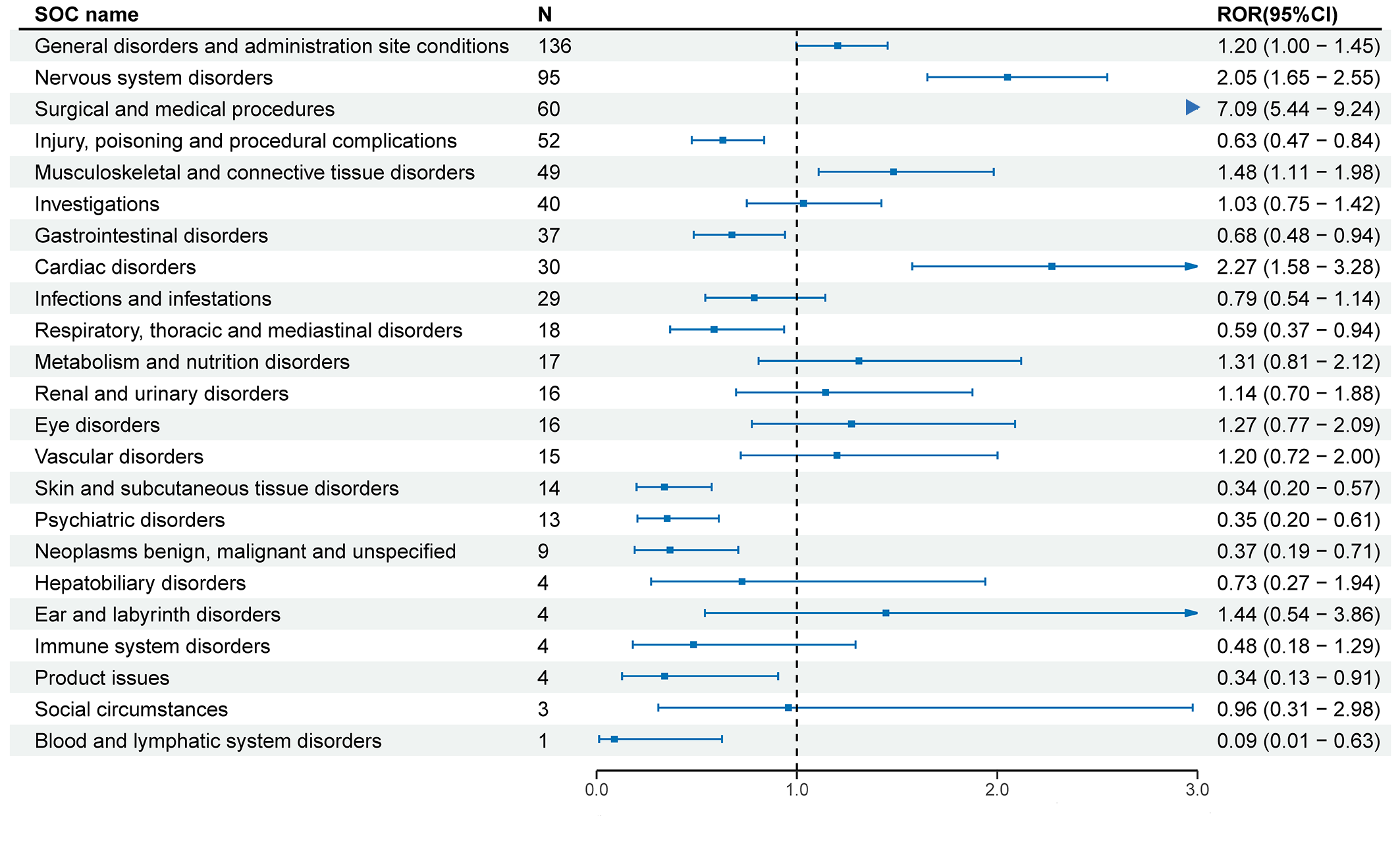


**Supplementary Figure 2**:Signal strength of AEs of Vutrisiran at the System Organ Class (SOC) level in FDA Adverse Event Reporting System (FAERS)


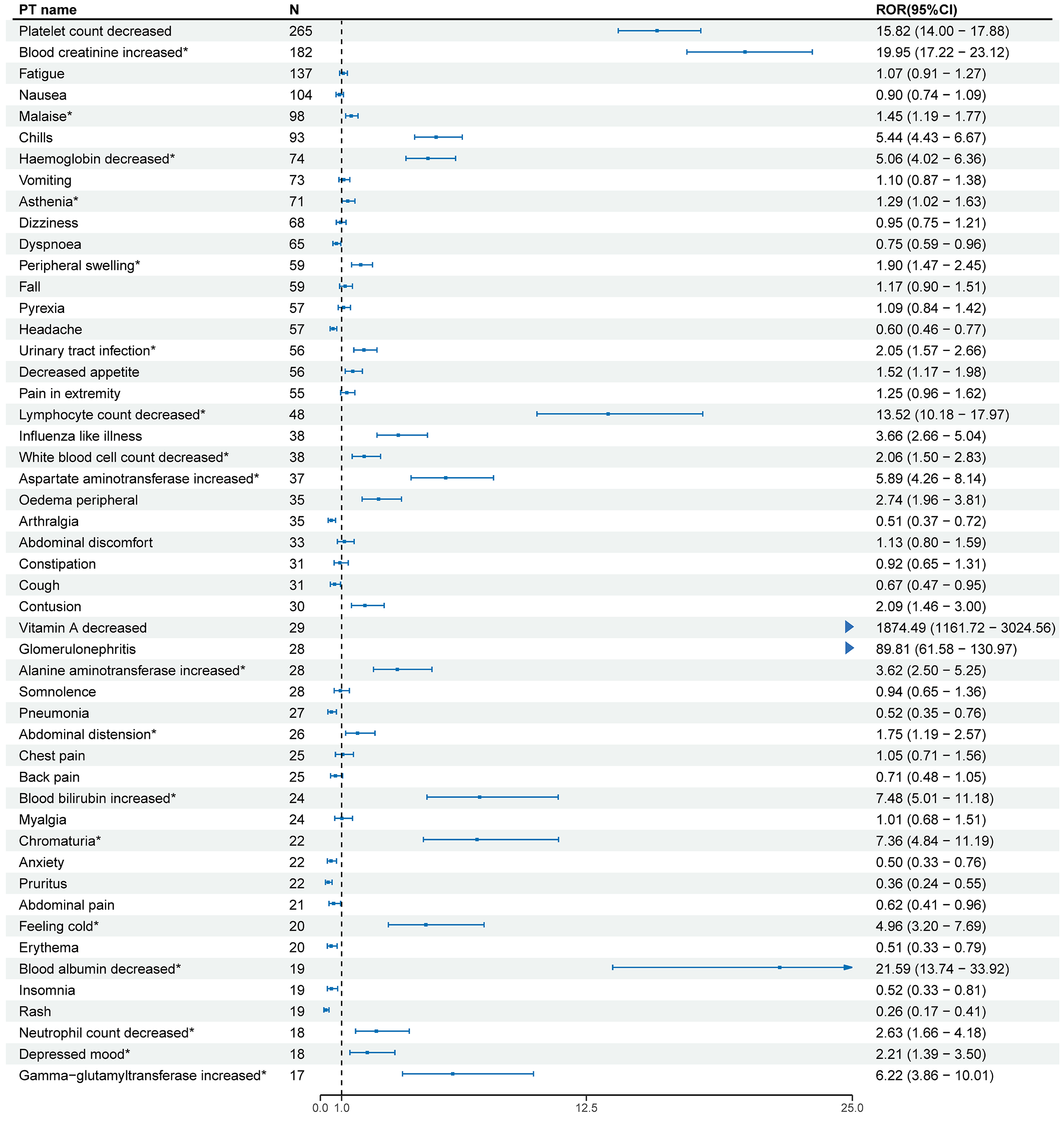


**Supplementary Figure 3**:Signal strength of AEs of Inotersen at the System Organ Class (SOC) level in FDA Adverse Event Reporting System (FAERS)

## Supplementary Tables

| Algorithms | Equation | Criteria |
| --- | --- | --- |
| ROR | ROR = ad/bc  95%CI = e^ln(ROR)±1^.^96(1/a+1/b+1/c+1/d)∧0^.^5^ | lower limit of 95%  CI > 1, a ≥ 2 |

**Supplementary Table 1**:Summary of algorithms used for signal detection.

a, number of reports containing both the suspect drug and the suspect adverse drug reaction; b, number of reports containing the suspect adverse drug reaction with other medications (except the drug of interest); c, number of reports containing the suspect drug with other adverse drug reactions (except the event of interest); d, number of reports containing other medications and other adverse drug reactions. ROR, reporting odds ratio。

| Subgroup | PT name | N | ROR | ROR025 | ROR975 |
| --- | --- | --- | --- | --- | --- |
| Medical professionals | Fatigue | 74 | 1.87 | 1.49 | 2.36 |
|  | Asthenia | 48 | 2.62 | 1.97 | 3.49 |
|  | Vomiting | 46 | 1.79 | 1.34 | 2.39 |
|  | Malaise | 41 | 2.03 | 1.49 | 2.76 |
|  | Back Pain | 38 | 3.32 | 2.41 | 4.57 |
|  | Somnolence | 38 | 3.83 | 2.78 | 5.27 |
|  | Nausea | 38 | 0.91 | 0.66 | 1.25 |
|  | Fall | 37 | 2.22 | 1.61 | 3.07 |
|  | Diarrhoea | 36 | 0.91 | 0.65 | 1.26 |
|  | Dyspnoea | 33 | 1.04 | 0.73 | 1.46 |
|  | Insomnia | 29 | 3.12 | 2.16 | 4.50 |
|  | Urinary Tract Infection | 28 | 2.86 | 1.97 | 4.16 |
|  | Pain In Extremity | 27 | 2.04 | 1.40 | 2.99 |
|  | Dizziness | 27 | 1.22 | 0.84 | 1.79 |
|  | Pyrexia | 27 | 1.13 | 0.78 | 1.65 |
|  | Headache | 26 | 0.87 | 0.59 | 1.28 |
|  | Pneumonia | 22 | 1.00 | 0.65 | 1.51 |
|  | Peripheral Swelling | 19 | 1.88 | 1.19 | 2.94 |
|  | Flushing | 17 | 3.93 | 2.44 | 6.33 |
|  | Cerebrovascular Accident | 16 | 2.87 | 1.75 | 4.68 |
|  | Chest Pain | 15 | 1.71 | 1.03 | 2.85 |
|  | Seizure | 15 | 1.74 | 1.05 | 2.89 |
|  | Syncope | 14 | 2.21 | 1.31 | 3.73 |
|  | Cough | 12 | 0.81 | 0.46 | 1.43 |
|  | Erythema | 12 | 1.18 | 0.67 | 2.07 |
|  | Abdominal Pain Upper | 12 | 1.34 | 0.76 | 2.36 |
|  | Decreased Appetite | 12 | 0.93 | 0.53 | 1.64 |
|  | Loss Of Consciousness | 11 | 1.76 | 0.97 | 3.18 |
|  | Muscle Spasms | 10 | 1.23 | 0.66 | 2.29 |
|  | Pleural Effusion | 10 | 2.32 | 1.25 | 4.32 |
|  | Abdominal Pain | 9 | 0.63 | 0.33 | 1.21 |
|  | Anxiety | 9 | 0.95 | 0.49 | 1.82 |
|  | Arthralgia | 9 | 0.38 | 0.20 | 0.73 |
|  | Restlessness | 9 | 4.81 | 2.50 | 9.26 |
|  | Arrhythmia | 9 | 3.40 | 1.77 | 6.54 |
|  | Rash | 9 | 0.32 | 0.17 | 0.61 |
|  | Dysphonia | 9 | 3.52 | 1.83 | 6.77 |
|  | Sepsis | 9 | 1.05 | 0.54 | 2.01 |
|  | Abdominal Discomfort | 8 | 0.85 | 0.42 | 1.70 |
|  | Vitamin A Decreased | 8 | 831.13 | 384.48 | 1796.65 |
|  | Pulmonary Oedema | 8 | 3.08 | 1.54 | 6.16 |
|  | Influenza Like Illness | 8 | 2.46 | 1.23 | 4.93 |
|  | Thrombosis | 8 | 2.05 | 1.02 | 4.10 |
|  | Hiccups | 7 | 17.57 | 8.35 | 36.94 |
|  | Chills | 7 | 1.08 | 0.51 | 2.26 |
|  | Nasopharyngitis | 7 | 0.73 | 0.35 | 1.54 |
|  | Urticaria | 7 | 0.71 | 0.34 | 1.49 |
|  | Septic Shock | 7 | 1.75 | 0.83 | 3.67 |
|  | Joint Swelling | 6 | 0.70 | 0.32 | 1.56 |
|  | Restless Legs Syndrome | 6 | 7.41 | 3.32 | 16.51 |
| Non-medical professionals | Fatigue | 60 | 1.57 | 1.22 | 2.03 |
|  | Fall | 42 | 2.94 | 2.17 | 3.99 |
|  | Asthenia | 36 | 2.30 | 1.65 | 3.19 |
|  | Nausea | 35 | 1.15 | 0.82 | 1.61 |
|  | Dizziness | 32 | 1.48 | 1.05 | 2.10 |
|  | Dyspnoea | 31 | 1.40 | 0.99 | 2.00 |
|  | Pain In Extremity | 27 | 1.96 | 1.34 | 2.87 |
|  | Pain | 26 | 0.62 | 0.42 | 0.92 |
|  | Back Pain | 25 | 2.45 | 1.65 | 3.63 |
|  | Somnolence | 24 | 2.87 | 1.92 | 4.28 |
|  | Malaise | 24 | 1.14 | 0.77 | 1.71 |
|  | Vomiting | 23 | 1.44 | 0.95 | 2.17 |
|  | Insomnia | 21 | 1.68 | 1.09 | 2.58 |
|  | Peripheral Swelling | 20 | 2.20 | 1.42 | 3.42 |
|  | Cerebrovascular Accident | 19 | 3.11 | 1.98 | 4.88 |
|  | Headache | 17 | 0.59 | 0.37 | 0.96 |
|  | Muscle Spasms | 16 | 2.01 | 1.23 | 3.28 |
|  | Urinary Tract Infection | 16 | 2.27 | 1.39 | 3.71 |
|  | Muscular Weakness | 14 | 3.12 | 1.84 | 5.27 |
|  | Arthralgia | 13 | 0.69 | 0.40 | 1.18 |
|  | Loss Of Consciousness | 13 | 2.88 | 1.67 | 4.96 |
|  | Chest Pain | 13 | 2.13 | 1.24 | 3.67 |
|  | Pneumonia | 13 | 1.16 | 0.67 | 2.01 |
|  | Decreased Appetite | 11 | 1.08 | 0.60 | 1.95 |
|  | Abdominal Pain Upper | 11 | 1.13 | 0.63 | 2.04 |
|  | Cough | 11 | 0.80 | 0.44 | 1.45 |
|  | Anxiety | 8 | 0.49 | 0.24 | 0.97 |
|  | Syncope | 8 | 2.70 | 1.35 | 5.40 |
|  | Sepsis | 7 | 2.83 | 1.35 | 5.94 |
|  | Vitamin A Decreased | 7 | 1667.47 | 704.49 | 3946.79 |
|  | Vertigo | 7 | 2.74 | 1.30 | 5.75 |
|  | Pyrexia | 7 | 0.69 | 0.33 | 1.45 |
|  | Depression | 7 | 0.70 | 0.33 | 1.47 |
|  | Seizure | 7 | 1.21 | 0.58 | 2.54 |
|  | Pruritus | 6 | 0.35 | 0.16 | 0.79 |
|  | Flushing | 6 | 1.99 | 0.89 | 4.43 |
|  | Limb Discomfort | 6 | 3.05 | 1.37 | 6.80 |
|  | Erythema | 6 | 0.44 | 0.20 | 0.99 |
|  | Infection | 6 | 1.21 | 0.54 | 2.69 |
|  | Dysphagia | 6 | 1.90 | 0.85 | 4.24 |
|  | Abdominal Pain | 6 | 0.82 | 0.37 | 1.82 |
|  | Abdominal Discomfort | 6 | 0.69 | 0.31 | 1.55 |
|  | Thrombosis | 6 | 1.66 | 0.74 | 3.69 |
|  | Chills | 5 | 1.15 | 0.48 | 2.75 |
|  | Sleep Disorder | 5 | 1.49 | 0.62 | 3.58 |
|  | Speech Disorder | 5 | 2.09 | 0.87 | 5.02 |
|  | Influenza | 5 | 0.90 | 0.37 | 2.17 |
|  | Taste Disorder | 5 | 3.18 | 1.32 | 7.64 |
|  | Memory Impairment | 5 | 0.60 | 0.25 | 1.45 |
|  | Abdominal Distension | 5 | 1.11 | 0.46 | 2.66 |

**Supplementary Table 2**:The top 50 AEs of Patisiran to Medical professionals and Non-medical professionals at preferred terms (PTs) level ranked by Case Numbers in FDA Adverse Event Reporting System (FAERS)

| Subgroup | PT name | N | ROR | ROR025 | ROR975 |
| --- | --- | --- | --- | --- | --- |
| Medical professionals | Hepatic Enzyme Increased | 4 | 17.27 | 6.40 | 46.55 |
|  | Sepsis | 4 | 9.96 | 3.69 | 26.84 |
|  | Malaise | 3 | 3.13 | 1.00 | 9.79 |
|  | Pain In Extremity | 3 | 4.81 | 1.54 | 15.07 |
|  | Abdominal Pain | 3 | 4.46 | 1.42 | 13.98 |
|  | Pain | 2 | 1.65 | 0.41 | 6.65 |
|  | Syncope | 2 | 6.66 | 1.65 | 26.85 |
|  | Rash | 2 | 1.49 | 0.37 | 6.02 |
|  | Asthenia | 2 | 2.29 | 0.57 | 9.22 |
|  | Arthralgia | 2 | 1.79 | 0.44 | 7.22 |
|  | Diarrhoea | 2 | 1.06 | 0.26 | 4.26 |
|  | Back Pain | 2 | 3.66 | 0.91 | 14.77 |
|  | Transaminases Increased | 1 | 9.10 | 1.27 | 65.01 |
|  | Haematuria | 1 | 8.55 | 1.20 | 61.04 |
|  | Gastric Ulcer Haemorrhage | 1 | 73.68 | 10.31 | 526.60 |
|  | Gastric Ulcer | 1 | 19.00 | 2.66 | 135.73 |
|  | Neoplasm | 1 | 35.94 | 5.03 | 256.78 |
|  | Pleural Effusion | 1 | 4.88 | 0.68 | 34.87 |
|  | Chills | 1 | 3.24 | 0.45 | 23.14 |
|  | Gastric Cancer | 1 | 15.59 | 2.18 | 111.32 |
|  | Pruritus | 1 | 1.01 | 0.14 | 7.24 |
|  | Gastrointestinal Disorder | 1 | 3.04 | 0.43 | 21.71 |
|  | Nausea | 1 | 0.50 | 0.07 | 3.56 |
|  | Vomiting | 1 | 0.81 | 0.11 | 5.78 |
|  | Pyrexia | 1 | 0.88 | 0.12 | 6.27 |
|  | Deep Vein Thrombosis | 1 | 5.84 | 0.82 | 41.68 |
|  | Oral Infection | 1 | 108.69 | 15.20 | 777.19 |
|  | Thrombosis | 1 | 5.39 | 0.75 | 38.48 |
|  | Cough | 1 | 1.42 | 0.20 | 10.13 |
|  | Muscle Spasms | 1 | 2.59 | 0.36 | 18.49 |
|  | Decreased Appetite | 1 | 1.63 | 0.23 | 11.66 |
|  | Electrolyte Imbalance | 1 | 21.66 | 3.03 | 154.68 |
|  | Liver Disorder | 1 | 8.28 | 1.16 | 59.12 |
|  | Lethargy | 1 | 7.93 | 1.11 | 56.66 |
|  | Hepatic Cirrhosis | 1 | 18.60 | 2.60 | 132.84 |
|  | Marasmus | 1 | 140.80 | 19.68 | 1007.25 |
|  | Cerebrovascular Accident | 1 | 3.76 | 0.53 | 26.83 |
|  | Haemoptysis | 1 | 11.72 | 1.64 | 83.68 |
|  | Haematemesis | 1 | 12.27 | 1.72 | 87.66 |
|  | Dizziness | 1 | 0.95 | 0.13 | 6.78 |
|  | Acute Kidney Injury | 1 | 1.18 | 0.17 | 8.42 |
|  | Pyelonephritis | 1 | 28.69 | 4.02 | 204.92 |
|  | Coma | 1 | 5.25 | 0.74 | 37.52 |
|  | Fall | 1 | 1.25 | 0.18 | 8.94 |
|  | Haematoma | 1 | 11.65 | 1.63 | 83.18 |
|  | Cerebral Haemorrhage | 1 | 8.29 | 1.16 | 59.21 |
|  | Renal Impairment | 1 | 3.11 | 0.44 | 22.24 |
|  | Myalgia | 1 | 2.45 | 0.34 | 17.48 |
|  | Blood Creatine Phosphokinase Abnormal | 1 | 663.79 | 92.15 | 4781.27 |
|  | Speech Disorder | 1 | 10.04 | 1.41 | 71.72 |
| Non-medical professionals | Fall | 13 | 4.88 | 2.81 | 8.46 |
|  | Pain In Extremity | 11 | 4.30 | 2.36 | 7.81 |
|  | Dyspnoea | 8 | 1.93 | 0.96 | 3.89 |
|  | Asthenia | 8 | 2.72 | 1.35 | 5.46 |
|  | Malaise | 7 | 1.78 | 0.84 | 3.76 |
|  | Loss Of Consciousness | 6 | 7.10 | 3.17 | 15.88 |
|  | Fatigue | 6 | 0.83 | 0.37 | 1.85 |
|  | Arthralgia | 5 | 1.41 | 0.58 | 3.40 |
|  | Dizziness | 5 | 1.23 | 0.51 | 2.96 |
|  | Peripheral Swelling | 5 | 2.93 | 1.21 | 7.07 |
|  | Hyperhidrosis | 4 | 4.55 | 1.70 | 12.16 |
|  | Vomiting | 4 | 1.32 | 0.50 | 3.54 |
|  | Back Pain | 4 | 2.08 | 0.78 | 5.56 |
|  | Urinary Tract Infection | 4 | 3.02 | 1.13 | 8.08 |
|  | Headache | 3 | 0.56 | 0.18 | 1.73 |
|  | Head Injury | 3 | 11.58 | 3.72 | 36.04 |
|  | Decreased Appetite | 3 | 1.57 | 0.50 | 4.87 |
|  | Nausea | 3 | 0.52 | 0.17 | 1.62 |
|  | Pneumonia | 3 | 1.43 | 0.46 | 4.44 |
|  | Renal Disorder | 3 | 6.60 | 2.12 | 20.54 |
|  | Limb Discomfort | 3 | 8.13 | 2.61 | 25.30 |
|  | Abdominal Pain | 2 | 1.45 | 0.36 | 5.80 |
|  | Blood Glucose Decreased | 2 | 4.21 | 1.05 | 16.88 |
|  | Pneumonia Aspiration | 2 | 25.58 | 6.38 | 102.65 |
|  | Subdural Haematoma | 2 | 44.32 | 11.04 | 177.92 |
|  | Sinusitis | 2 | 2.57 | 0.64 | 10.30 |
|  | Acute Kidney Injury | 2 | 2.37 | 0.59 | 9.49 |
|  | Seizure | 2 | 1.84 | 0.46 | 7.36 |
|  | Coma | 2 | 12.28 | 3.06 | 49.24 |
|  | Contusion | 2 | 2.36 | 0.59 | 9.48 |
|  | Syncope | 2 | 3.58 | 0.89 | 14.36 |
|  | Depressed Mood | 2 | 4.02 | 1.00 | 16.11 |
|  | Hypersomnia | 2 | 7.03 | 1.75 | 28.21 |
|  | Confusional State | 2 | 2.01 | 0.50 | 8.06 |
|  | Mental Impairment | 2 | 9.16 | 2.28 | 36.74 |
|  | Cerebrovascular Accident | 2 | 1.73 | 0.43 | 6.94 |
|  | Anxiety | 2 | 0.65 | 0.16 | 2.59 |
|  | Chest Pain | 1 | 0.87 | 0.12 | 6.17 |
|  | Pulmonary Embolism | 1 | 3.10 | 0.44 | 22.08 |
|  | Pulmonary Oedema | 1 | 3.76 | 0.53 | 26.77 |
|  | Upper Gastrointestinal Haemorrhage | 1 | 10.47 | 1.47 | 74.54 |
|  | Limb Injury | 1 | 3.24 | 0.46 | 23.06 |
|  | Infection | 1 | 1.07 | 0.15 | 7.60 |
|  | Vitamin A Decreased | 1 | 972.26 | 131.68 | 7178.89 |
|  | Vertigo | 1 | 2.08 | 0.29 | 14.77 |
|  | Blindness | 1 | 2.39 | 0.34 | 16.98 |
|  | Subdural Haemorrhage | 1 | 138.89 | 19.42 | 993.32 |
|  | Anaemia | 1 | 1.39 | 0.20 | 9.87 |
|  | Early Satiety | 1 | 104.89 | 14.69 | 749.15 |
|  | Gastrointestinal Motility Disorder | 1 | 20.32 | 2.85 | 144.65 |

**Supplementary Table 3**:The top 50 AEs of Vutrisiran to Medical professionals and Non-medical professionals at preferred terms (PTs) level ranked by Case Numbers in FDA Adverse Event Reporting System (FAERS)

| Subgroup | PT name | N | ROR | ROR025 | ROR975 |
| --- | --- | --- | --- | --- | --- |
| Medical professionals | Platelet Count Decreased | 182 | 12.83 | 11.07 | 14.87 |
|  | Blood Creatinine Increased | 129 | 14.88 | 12.49 | 17.72 |
|  | Fatigue | 89 | 1.10 | 0.89 | 1.36 |
|  | Malaise | 67 | 1.63 | 1.28 | 2.07 |
|  | Nausea | 67 | 0.79 | 0.62 | 1.00 |
|  | Haemoglobin Decreased | 61 | 5.11 | 3.97 | 6.58 |
|  | Chills | 52 | 3.97 | 3.02 | 5.22 |
|  | Dizziness | 49 | 1.09 | 0.83 | 1.45 |
|  | Vomiting | 47 | 0.89 | 0.67 | 1.19 |
|  | Pain | 47 | 0.91 | 0.68 | 1.21 |
|  | Asthenia | 46 | 1.23 | 0.92 | 1.64 |
|  | Urinary Tract Infection | 41 | 2.06 | 1.52 | 2.80 |
|  | Fall | 41 | 1.21 | 0.89 | 1.64 |
|  | Dyspnoea | 41 | 0.63 | 0.46 | 0.86 |
|  | Decreased Appetite | 41 | 1.57 | 1.16 | 2.14 |
|  | Lymphocyte Count Decreased | 40 | 10.91 | 7.99 | 14.90 |
|  | Headache | 39 | 0.64 | 0.47 | 0.88 |
|  | Peripheral Swelling | 37 | 1.80 | 1.30 | 2.49 |
|  | Pain In Extremity | 37 | 1.38 | 1.00 | 1.90 |
|  | Pyrexia | 35 | 0.72 | 0.52 | 1.01 |
|  | Aspartate Aminotransferase Increased | 33 | 4.34 | 3.09 | 6.12 |
|  | White Blood Cell Count Decreased | 30 | 1.86 | 1.30 | 2.66 |
|  | Influenza Like Illness | 25 | 3.80 | 2.56 | 5.63 |
|  | Arthralgia | 25 | 0.52 | 0.35 | 0.77 |
|  | Alanine Aminotransferase Increased | 24 | 2.55 | 1.71 | 3.81 |
|  | Abdominal Discomfort | 24 | 1.26 | 0.84 | 1.88 |
|  | Glomerulonephritis | 23 | 64.40 | 42.45 | 97.70 |
|  | Cough | 23 | 0.76 | 0.51 | 1.15 |
|  | Vitamin A Decreased | 22 | 1915.54 | 1044.99 | 3511.33 |
|  | Blood Bilirubin Increased | 22 | 6.03 | 3.96 | 9.16 |
|  | Abdominal Distension | 20 | 2.14 | 1.38 | 3.32 |
|  | Back Pain | 20 | 0.85 | 0.55 | 1.32 |
|  | Pneumonia | 18 | 0.40 | 0.25 | 0.64 |
|  | Contusion | 18 | 2.08 | 1.31 | 3.30 |
|  | Myalgia | 18 | 1.03 | 0.65 | 1.64 |
|  | Somnolence | 18 | 0.89 | 0.56 | 1.41 |
|  | Swelling | 17 | 1.38 | 0.85 | 2.21 |
|  | Pruritus | 17 | 0.40 | 0.25 | 0.65 |
|  | Blood Albumin Decreased | 16 | 17.09 | 10.44 | 27.97 |
|  | Blood Lactate Dehydrogenase Increased | 16 | 7.62 | 4.66 | 12.46 |
|  | Neutrophil Count Decreased | 15 | 1.88 | 1.13 | 3.12 |
|  | Gamma-Glutamyltransferase Increased | 15 | 4.46 | 2.69 | 7.41 |
|  | Abdominal Pain Upper | 14 | 0.77 | 0.45 | 1.30 |
|  | Blood Alkaline Phosphatase Increased | 14 | 4.49 | 2.65 | 7.58 |
|  | Anxiety | 14 | 0.73 | 0.43 | 1.23 |
|  | Erythema | 14 | 0.68 | 0.40 | 1.14 |
|  | Feeling Cold | 14 | 6.01 | 3.55 | 10.15 |
|  | Depressed Mood | 14 | 2.94 | 1.74 | 4.98 |
|  | Abdominal Pain | 13 | 0.45 | 0.26 | 0.77 |
|  | Chest Pain | 12 | 0.67 | 0.38 | 1.19 |
| Non-medical professionals | Platelet Count Decreased | 83 | 21.84 | 17.54 | 27.18 |
|  | Blood Creatinine Increased | 53 | 30.39 | 23.14 | 39.92 |
|  | Fatigue | 48 | 1.23 | 0.92 | 1.64 |
|  | Chills | 41 | 9.37 | 6.88 | 12.77 |
|  | Nausea | 37 | 1.20 | 0.87 | 1.66 |
|  | Malaise | 31 | 1.46 | 1.02 | 2.08 |
|  | Pain | 30 | 0.71 | 0.49 | 1.02 |
|  | Vomiting | 26 | 1.60 | 1.09 | 2.35 |
|  | Asthenia | 25 | 1.56 | 1.05 | 2.32 |
|  | Dyspnoea | 24 | 1.07 | 0.71 | 1.59 |
|  | Pyrexia | 22 | 2.14 | 1.41 | 3.26 |
|  | Peripheral Swelling | 22 | 2.38 | 1.57 | 3.62 |
|  | Dizziness | 19 | 0.86 | 0.55 | 1.35 |
|  | Fall | 18 | 1.23 | 0.77 | 1.95 |
|  | Headache | 18 | 0.62 | 0.39 | 0.98 |
|  | Pain In Extremity | 18 | 1.28 | 0.81 | 2.04 |
|  | Urinary Tract Infection | 15 | 2.09 | 1.26 | 3.47 |
|  | Decreased Appetite | 15 | 1.45 | 0.87 | 2.41 |
|  | Haemoglobin Decreased | 13 | 3.72 | 2.15 | 6.41 |
|  | Chest Pain | 13 | 2.09 | 1.21 | 3.61 |
|  | Influenza Like Illness | 13 | 4.05 | 2.35 | 6.98 |
|  | Contusion | 12 | 2.63 | 1.49 | 4.63 |
|  | Arthralgia | 10 | 0.52 | 0.28 | 0.96 |
|  | Somnolence | 10 | 1.17 | 0.63 | 2.17 |
|  | Memory Impairment | 10 | 1.18 | 0.64 | 2.20 |
|  | Rash | 10 | 0.54 | 0.29 | 1.01 |
|  | Pneumonia | 9 | 0.79 | 0.41 | 1.52 |
|  | Red Blood Cell Count Decreased | 9 | 6.90 | 3.58 | 13.27 |
|  | Abdominal Discomfort | 9 | 1.02 | 0.53 | 1.97 |
|  | Cough | 8 | 0.57 | 0.29 | 1.14 |
|  | Anaemia | 8 | 2.06 | 1.03 | 4.12 |
|  | Lymphocyte Count Decreased | 8 | 13.96 | 6.97 | 27.97 |
|  | Anxiety | 8 | 0.48 | 0.24 | 0.96 |
|  | Insomnia | 8 | 0.63 | 0.31 | 1.25 |
|  | Abdominal Pain | 8 | 1.07 | 0.53 | 2.14 |
|  | White Blood Cell Count Decreased | 8 | 1.98 | 0.99 | 3.97 |
|  | Aspartate Aminotransferase Abnormal | 8 | 258.42 | 126.69 | 527.12 |
|  | Loss Of Consciousness | 8 | 1.74 | 0.87 | 3.48 |
|  | Vitamin A Decreased | 7 | 1638.76 | 692.37 | 3878.78 |
|  | Confusional State | 7 | 1.30 | 0.62 | 2.73 |
|  | Sleep Disorder | 7 | 2.05 | 0.98 | 4.30 |
|  | Syncope | 6 | 1.98 | 0.89 | 4.42 |
|  | Abdominal Distension | 6 | 1.31 | 0.59 | 2.91 |
|  | Erythema | 6 | 0.44 | 0.20 | 0.97 |
|  | Myalgia | 6 | 0.94 | 0.42 | 2.09 |
|  | Muscle Spasms | 6 | 0.74 | 0.33 | 1.64 |
|  | Alanine Aminotransferase Abnormal | 6 | 139.71 | 61.97 | 314.97 |
|  | Pulmonary Oedema | 6 | 4.18 | 1.88 | 9.32 |
|  | Feeling Cold | 6 | 4.55 | 2.04 | 10.13 |
|  | Acute Kidney Injury | 6 | 1.31 | 0.59 | 2.92 |

**Supplementary Table 4**:The top 50 AEs of Inotersen to Medical professionals and Non-medical professionals at preferred terms (PTs) level ranked by Case Numbers in FDA Adverse Event Reporting System (FAERS)
